# The ‘critical trochanter angle’: a predictor for stem alignment in total hip arthroplasty

**DOI:** 10.1186/s13018-019-1206-x

**Published:** 2019-05-30

**Authors:** Marcel Haversath, André Busch, Marcus Jäger, Tjark Tassemeier, Daniel Brandenburger, Sebastian Serong

**Affiliations:** 1 Department of Orthopaedics and Trauma Surgery, University of Duisburg-Essen, Hufelandstr. 55, 45147 Essen, Germany; 2 Department of Orthopaedics and Orthopaedic Surgery, Saarland University, Homburg, Germany

**Keywords:** Critical trochanter angle, Stem alignment, Direct anterior approach, Direct lateral approach, Total hip arthroplasty, Corail

## Abstract

**Introduction:**

Stem malalignment can affect offset reconstruction and may result in gluteal muscle insufficiency. In this retrospective study, a novel geometric angle named ‘critical trochanter angle’ (CTA) is described and investigated towards the risk of malposition of a collarless straight tapered hydroxyapatite-coated stem in primary total hip arthroplasty (THA).

**Material and methods:**

A total of 100 cementless THA were implanted in patients with unilateral coxarthrosis via the direct anterior (*n* = 50) or direct lateral Hardinge approach (*n* = 50) in a two surgeon setting using the Corail® or Trendhip® stem (DePuy Synthes or Aesculap). Stem alignment was analysed in postoperative AP pelvic radiographs and correlated to the CTA: the angle crest was defined by the intersection of the femoral shaft and neck axis and the angle was measured between the shaft axis and a leg intersecting the vertex between the lateral and superoposterior facet of the trochanter.

**Results:**

Forty-seven stems were implanted in varus (≥ + 1°), 42 in neutral (< + 1°/> − 1°) and 11 in valgus position (≤ − 1°). The mean critical trochanter angle was 25.0° (SD ± 7.5°), and there was a negative and statistically significant correlation to stem alignment (*r* = − 0.52; *p* ≤ 0.001) independent from the surgical approach. For stem malposition of 2° and above (*n* = 23), mean CTA was 17.2° for varus (*n* = 20) and 31.6° for valgus (*n* = 3). A CTA lesser or equal to 22.75° had a sensitivity of 90% and specificity of 80% for varus stem position of 2° or greater. Specificity raised to 100% with a cutoff CTA of 12.5° or lesser.

**Conclusion:**

Varus stem alignment in THA is associated with coxa vara deformity and a radiological low CTA. In preoperative planning, the critical trochanter angle can help to evaluate the risk for intraoperative stem malpositioning. If navigation or robotic assistance is not available when using this stem design, we recommend an intraoperative x-ray to verify correct implant positioning in patients with a CTA under 20° or above 30°.

## Background

Offset restoration in total hip arthroplasty (THA) is crucial for the lever arm and function of the gluteal muscles. In proper preoperative planning, stem position is usually placed neutrally and parallel to the femoral shaft axis. In contrast, intraoperative stem malpositioning in the coronal plane may affect offset or leg length restoration and can hamper optimum load transfer between the implant and the natural bone [1]. Recently, it was described that coxa vara deformity which includes a low centrum collum diaphyseal (CCD) angle, a long neck and a trochanter overhang enhances the risk for intraoperative varus stem positioning [2]. In this retrospective study, the coronal position of two similar collarless straight stem designs with a narrow shoulder (Corail®, DePuy Synthes and Trendhip®, Aesculap) was analysed. A novel geometric angle named ‘critical trochanter angle’ (CTA) was identified as a predictor for stem alignment. This angle measures the extent of the trochanter overhang in relation to the femoral shaft axis and will be firstly described and analysed in this study in detail.

## Material and methods

**Fig. 1:**
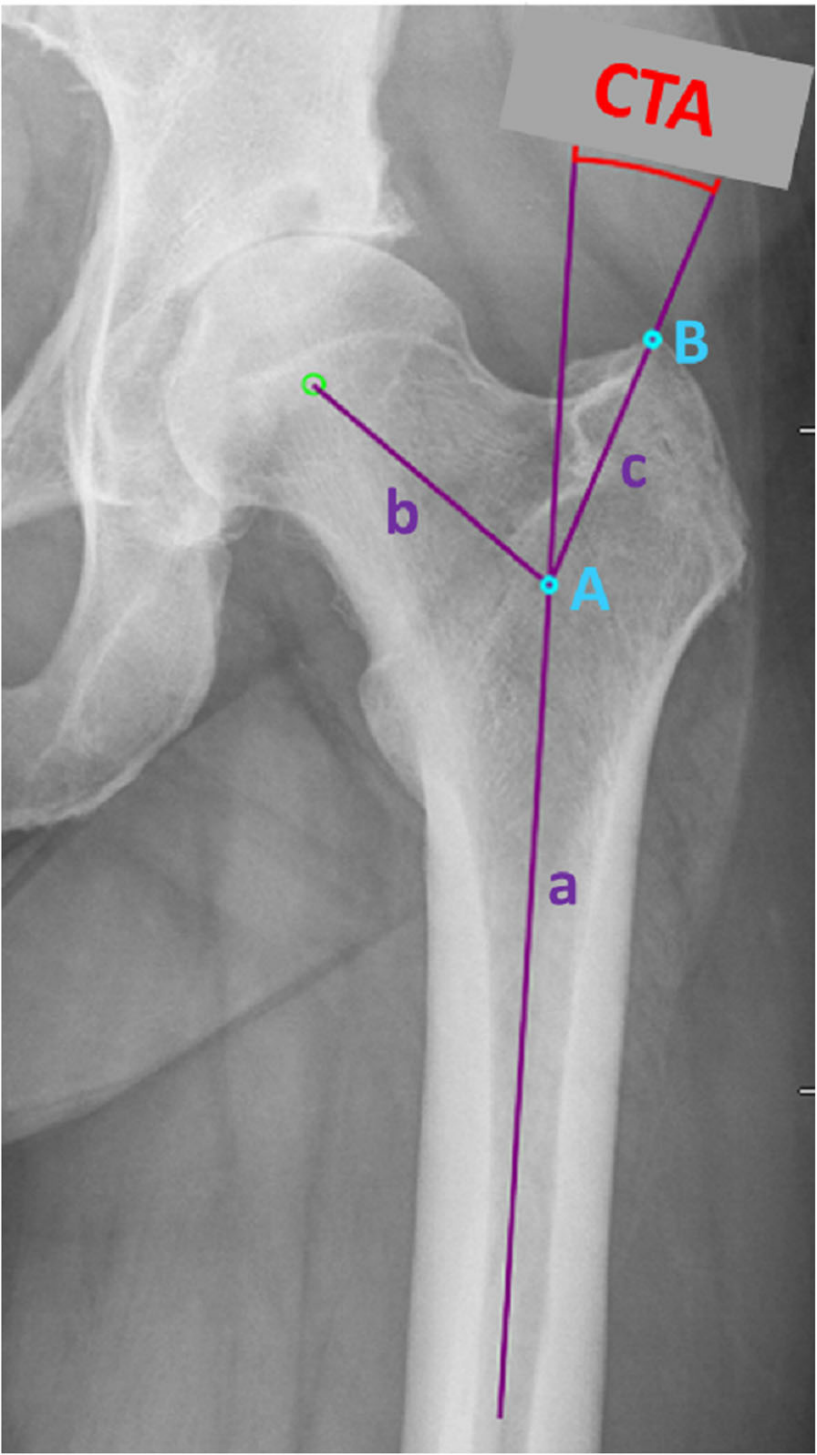
Measurement of the critical trochanter angle (CTA). The angle crest (A) is defined as the intersection of the femoral shaft axis (a) and the femoral neck axis (b). A leg (c) between the angle crest (A) and trochanter vertex (B) is build, and the CTA is measured between this leg and the femoral shaft axis

One hundred patients (male: *n* = 42, female: *n* = 58) with unilateral coxarthrosis (Kellgren and Lawrence ≥ 3) or osteonecrosis of the femoral head were retrospectively included in this study using a preoperative (up to 6 weeks before implantation) and a postoperative (1 week after THA) true anteroposterior (AP) pelvic radiograph. Exclusion criteria were a previous proximal femoral fracture or fractures occurring intra- or postoperatively (i.e. trochanter tip fractures). Furthermore, patients with abnormal proximal femoral deformities and trochanter nearby ossifications or patients that were not x-rayed in a true AP pelvic view were excluded. All patients received a cementless total hip replacement via the direct anterior approach (DAA; *n* = 50) in the technique described by Bender et al. [3] or direct lateral Hardinge approach (LAT; *n* = 50). A collarless straight tapered hydroxyapatite (HA)-coated stem (Corail® or Trendhip®) was combinated with a cementless hemispheric cup (Pinnacle®, Depuy Synthes or Plasmafit®, Aesculap) and ceramic-on-polyethylene bearing. In preoperative AP pelvic radiographs, the following parameters were measured using the mediCAD® planning software (mediCAD Hectec GmbH, Altdorf, Germany): ipsilateral femoral offset, CCD angle and the critical trochanter angle (CTA). The angle crest of the CTA was defined by the intersection of the femoral shaft and neck axis. Then, the angle was measured between the femoral shaft axis and a leg intersecting the vertex between the lateral and superoposterior facet of the trochanter (Fig. 1). In postoperative radiographs, stem alignment was measured and grouped in varus (≥ + 1°), neutral (< + 1°/> − 1°) and valgus position (≤ − 1°). Therefore, an angle between the femoral shaft axis and the stem axis was drawn and measured. Finally, stem alignment was correlated to femoral offset, CCD angle and CTA.

Descriptive and statistical analysis was performed using IBM® SPSS® Statistics, version 25. Pearson’s coefficient was used for the correlation of stem alignment and metric data (femoral offset/CCD/CTA), and a receiver operating characteristic (ROC) curve was obtained to analyse the sensitivity and specificity of CTA cutoff values that result in stem malalignment equal to or greater than 2°. Finally, the independent samples *t* test was used to compare the two surgical approaches towards stem alignment and Pearson’s coefficients were compared using Fisher’s *z*-transformation.

We postulate that the CTA is correlated with stem alignment in THA when using the abovementioned stem.

## Results

### Stem alignment

In preoperative planning, variances in stem alignment have a significant effect on femoral offset reconstruction. One degree deviation from the neutral axis may result in up to 3 mm offset shift when using one of the abovementioned stems in the largest size and high offset variant. In this retrospective analysis, 47 stems were implanted in varus (47% ≥ + 1°), 42 in neutral (< + 1°/> − 1°) and 11 in valgus position (≤ − 1°). Mean stem position in varus was + 2.2° (SD ± 1.2°) and in valgus − 1.7° (SD ± 0.6°). Alignment ranged from − 2.8° valgus to + 7.0° varus. The surgical approach had no significant influence on stem alignment (*p* = 0.12).

### The ‘critical trochanter angle’

**Fig. 2:**
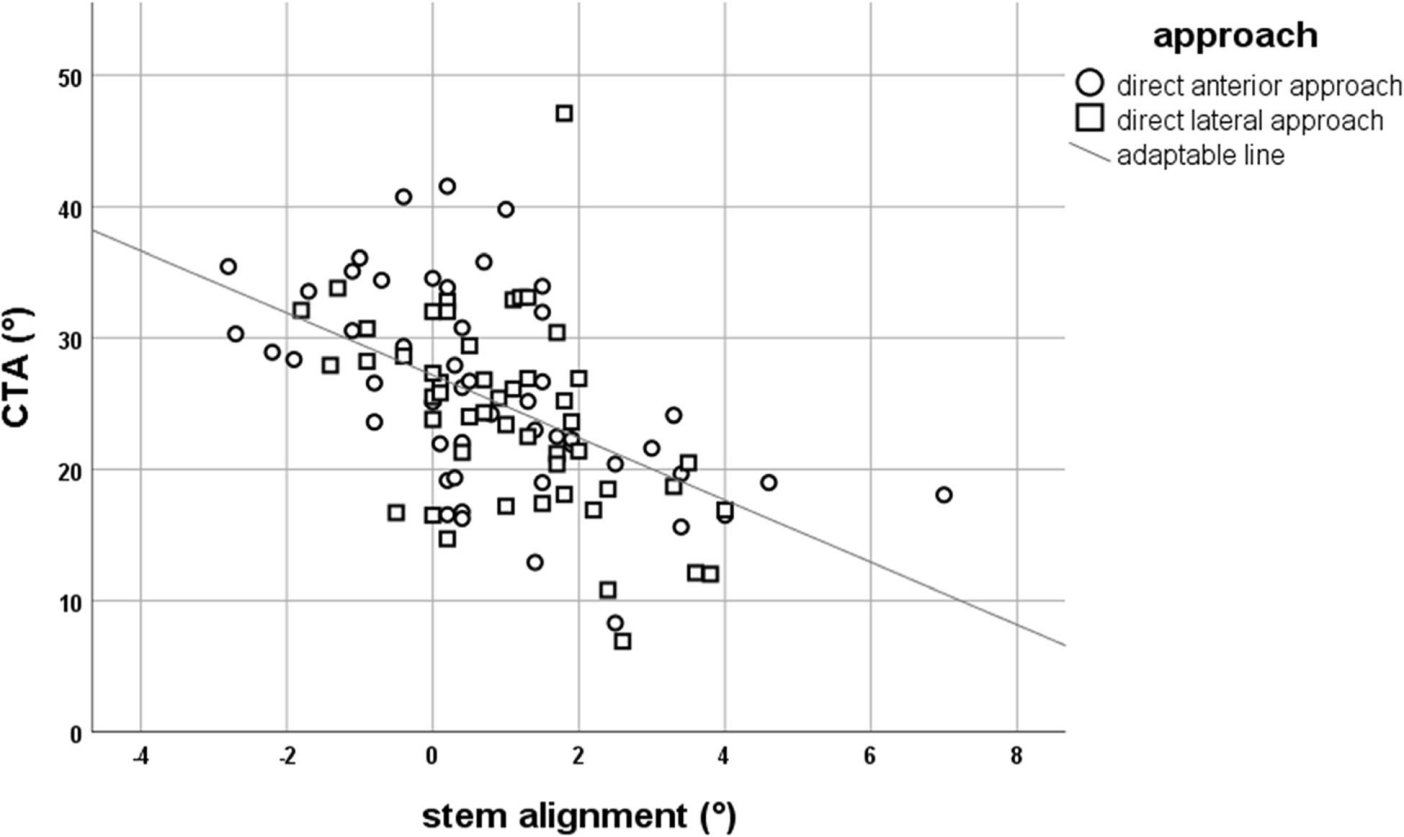
Distribution of stem alignment and CTA. A negative correlation is illustrated which seems to be independent from the surgical approach that was used for implantation

The CTA measures the trochanter overhang independent from the individual size of the hip. Therefore, a scaling of the pelvic radiographs is not necessary when using this angle to evaluate the risk of stem malpositioning. The mean CTA in this cohort was 25.0° (SD ± 7.5°). A low CTA was correlated with varus stem alignment, and a high CTA was found in patients with postoperative valgus stem position. This correlation was statistically significant (*r* = − 0.52; *p* ≤ 0.001). In the varus group, mean CTA was 22.2°; in the neutral group, it was 26.3°; and in the valgus group, it was 32.0°, respectively. The ROC curve analysis revealed that a CTA lesser or equal to 22.75° had a sensitivity of 90% and specificity of 80% for varus stem position of 2° or greater. On the other hand, a CTA above or equal to 35.63° had a sensitivity of 100% and specificity of 93.8% for valgus stem position of 2° or greater. Figure 2 demonstrates the distribution of stem alignment in dependency of the CTA.

### Analysis of femoral offset and centrum collum diaphyseal angle

In THA, femoral offset reconstruction is crucial for the lever arm of the pelvitrochanteric musculature. Mean preoperative femoral offset was 38.9 mm (SD ± 6.9 mm), and a significant correlation of femoral offset and stem alignment could be detected (*r* = 0.62; *p* ≤ 0.001) meaning that a high preoperative offset often resulted in varus stem positioning. Similarly, a low CCD correlated with varus alignment (*r* = − 0.46; *p* ≤ 0.001). This moderate downhill correlation of *r* = − 0.46 was slightly weaker than for the CTA and varus alignment (*r* = − 0.52), but the difference of both correlation coefficients was statistically not significant (*p* = 0.23). Overall mean CCD was 128.1° (SD ± 6.1°). In the varus group, it was 125.7°, in the neutral group 129.3° and in the valgus group 133.7°, respectively.

**Fig. 3:**
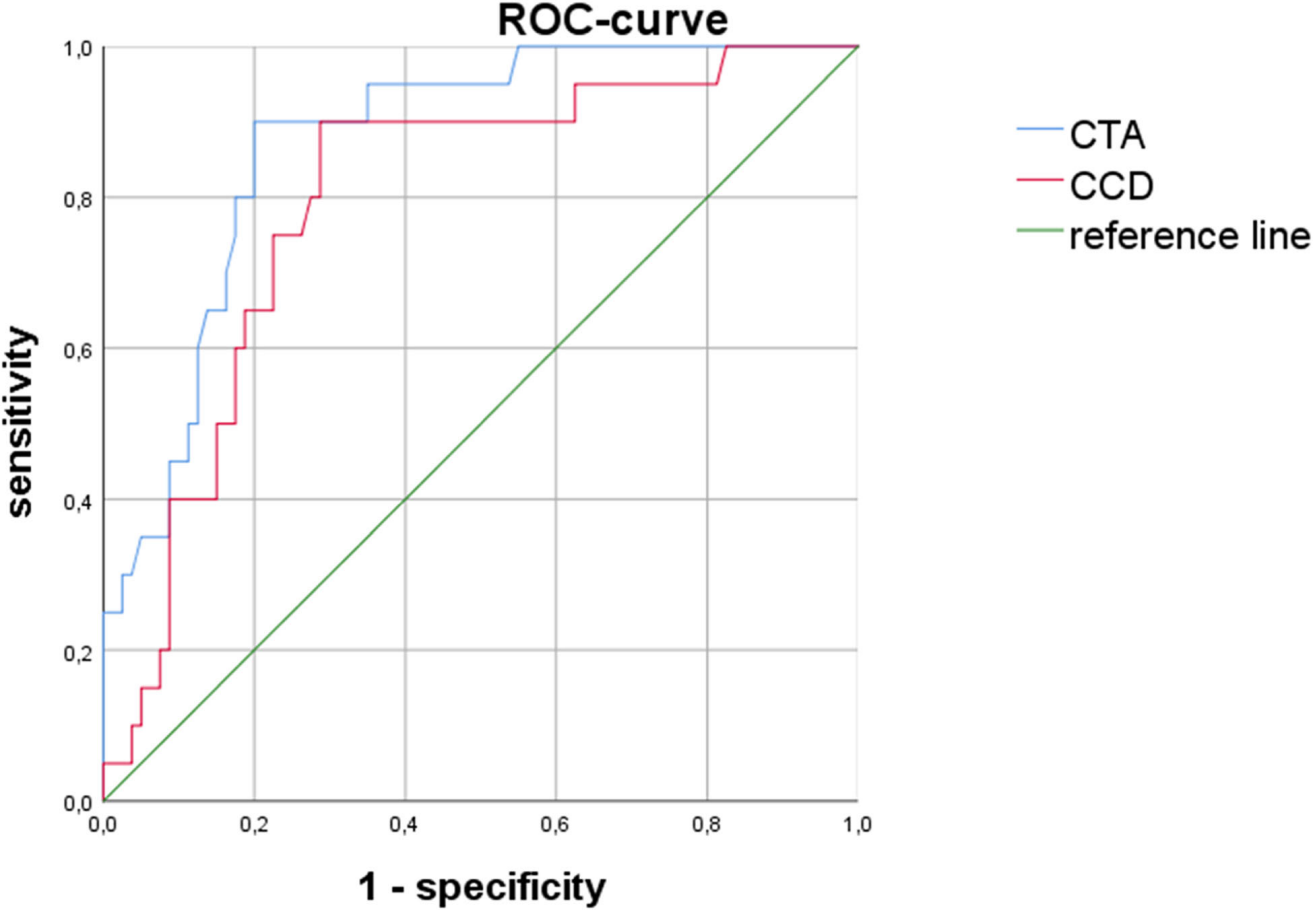
The ROC curve illustrates the sensitivity and specificity of the CTA compared to the CCD in cases of postoperatively measured varus stems of 2° and above (*n* = 20). The CTA seems to be more sensitive and specific for varus malalignment than the CCD

In conclusion, a previous coxa vara with a long neck was more likely to result in varus stem positioning and this risk was statistically relevant. A highly significant negative correlation of femoral offset and CTA as well as positive correlation of CCD and CTA could be observed (femoral offset and CTA: *r* = − 0.63, *p* ≤ 0.001; CCD and CTA: *r* = 0.54, *p* ≤ 0.001). However, the CCD is less sensitive and specific than CTA to predict varus stem positioning of 2° and above (Fig. 3).

## Discussion

Stem alignment in total hip arthroplasty is relevant not only for femoral offset restoration but also for permanent osseointegration of the implant [4]. Especially, varus stem alignment is frequently seen in coxa vara deformity and this relationship was recently described in the literature [2]. Implanting a straight tapered stem in varus alignment may result in poor clinical outcome. For example, it was shown that varus position can lead to femoral cortical hypertrophy and thigh pain [5] and it may negatively influence long-term survivorship [6]. In varus, a higher distal strain distribution compared to the neutral position seems to be the reason for those findings [1]. However, valgus stem alignment does not seem to be better than varus in terms of clinical outcome or long-term survival. A valgus position may enhance stress shielding at the proximal femur compared to varus [7] and is at higher risk to loose femoral offset compared to the preoperative status. A loss of femoral offset should be avoided as this shortens the lever arm and enhances the stress for the gluteal muscles. Therefore, Bjordal and Bjorgul suggest that the surgeon should aim for an equal or slightly increased lever arm [8]. The hydroxyapatite-coated straight tapered cementless stem which was analysed in this study is known to offer good primary stability and reliable osseointegration [9]. In a patient-specific finite element model, the Corail® stem is stable and robust to changes in position, even when undersized by up to two sizes [10]. Furthermore, long-term survivorship of the Corail® stem is more than satisfactory. Twelve years after implantation, a survival of 95% was described [11]. Others found 97% survival over 17 years [12]. However, the HA coating does not seem to be the reason for these good results. The Nordic Arthroplasty Register Association did not find a benefit of HA-coated cups or stems in terms of long-term survival [13, 14].

As described above, stem malalignment is a common finding in postoperative radiographs after THA. To better evaluate the preoperative risk for intraoperative malpositioning, we identified the critical trochanter angle that describes the trochanter overhang as a relative parameter and is independent from the intraindividual size of the hip. The rationale for this angle can be explained as follows: a low CTA describes a high trochanter overhang which inhibits lateral preparation towards the trochanter with the femoral rasp resulting in varus stem positioning. Vice versa, a high CTA describes a low trochanter overhang and can provoke an extended preparation towards the trochanter resulting in valgus stem alignment.

In the investigated cohort, a high incidence of varus stems could be observed (47%). We did not find a significant difference between both surgical approaches, and our results correspond to the findings of Batailler et al. who described a varus position in 40% when using the collared Corail® stem via the DAA [15]. This particular stem design might tend to malalignment, especially in coxa vara deformity [2]. The reason is not fully clear, but it was recently excluded that femoral neck resection height is associated with the final position of the Corail® stem [16]. However, the CTA was significantly correlated to the CCD and a significant negative correlation could be found to femoral offset. Therefore, the CTA is a parameter not only to evaluate the risk of stem malpositioning but also to describe hip deformity and it seems to be more sensitive and specific than the CCD.

Limitations of this study include a lack of clinical data such as the body mass index. These clinical parameters may have an effect on intraoperative preparation of the femur and subsequently on stem alignment. However, the sampling size was evaluated as high enough to reduce the effect of clinical confounder.

Finally, our hypothesis that stem alignment is associated with the CTA could be confirmed in this study. The CTA aims that surgeons pay preoperatively more attention on the trochanter overhang that may increase the risk for stem malalignment. The CTA is a useful angle to describe the trochanter overhang in comparison to the femoral shaft axis, and it is measured in degrees which makes it independent from the metric size of the hip.

The correlation of the CTA to the alignment of other stem designs as well as the intra- and interobserver reliability of this angle should be investigated in further prospective trials including analysis of clinical data.

## Conclusions

Varus stem alignment is associated with coxa vara deformity in THA. In preoperative planning, the critical trochanter angle can help to evaluate the risk for intraoperative stem malpositioning in the coronal plane. In case of a low CTA, extended preparation of the methaphysis towards the lateral trochanteric region is necessary to avoid varus stem implantation. If navigation or robotic assistance is not available when using this particular stem design, we recommend an intraoperative x-ray to verify correct stem positioning in patients with a CTA under 20° or above 30°.

## Data Availability

The datasets used and analysed during the current study are available from the corresponding author on reasonable request.

## Abbreviations

AP: Anteroposterior
CCD: Centrum collum diaphyseal
CTA: Critical trochanter angle
DAA: Direct anterior approach
HA: Hydroxyapatite
LAT: Direct lateral Hardinge approach
ROC: Receiver operating characteristic
THA: Total hip arthroplasty
